# Psychosocial risk and protective factors for the health and well-being of professionals working in emergency and non-emergency medical transport services, identified via questionnaires

**DOI:** 10.1186/s13049-017-0433-6

**Published:** 2017-09-06

**Authors:** P. Navarro Moya, M. González Carrasco, E. Villar Hoz

**Affiliations:** 1 Doctoral student in Psychology, Health and Quality of Life, University of Girona, Girona, Spain; 2 Doctor of Psychology, Institut de Recerca sobre Qualitat de Vida, University of Girona, Girona, Spain; 3 Doctor of Psychology, Departament de Psicologia, University of Girona, Girona, Spain

**Keywords:** Medical transport, Emergency health workers, Working conditions, Occupational health, Burnout, Sense of coherence, Subjective well-being

## Abstract

**Background:**

Medical transport (MT) professionals are subject to considerable emotional demands due to their involvement in life-or-death situations and their exposure to the serious health problems of their clients. An increase in the demand for MT services has, in turn, increased interest in the study of the psychosocial risk factors affecting the health of workers in this sector. However, research thus far has not distinguished between emergency (EMT) and non-emergency (non-EMT) services, nor between the sexes. Furthermore, little emphasis has been placed on the protective factors involved. The main objective of the present study is to identify any existing differential exposure – for reasons of work setting (EMT and non-EMT) or of gender – to the various psychosocial risk and protective factors affecting the health of MT workers.

**Methods:**

Descriptive and transversal research with responses from 201 professionals.

**Results:**

The scores obtained on the various psychosocial scales in our study – as indicators of future health problems – were more unfavourable for non-EMT workers than they were for EMT workers. Work setting, but not gender, was able to account for these differences.

**Discussion:**

The scores obtained for the different psychosocial factors are generally more favourable for the professionals we surveyed than those obtained in previous samples.

**Conclusion:**

The significant differences observed between EMT and non-EMT personnel raise important questions regarding the organization of work in companies that carry out both services at the same time in the same territory. The relationships among the set of risk/protective factors suggests a need for further investigation into working conditions as well as a consideration of the workers’ *sense of coherence* and subjective well-being as protective factors against occupational burnout syndrome.

## Background

### The psychosocial health of medical transport professionals

Medical transport (MT) has undergone significant growth in recent years [1, 2]. Terrestrial MT can be divided into two types: emergency (EMT) and non-emergency (non-EMT). The increased demand and the quality of service required for both these types of medical transport has stimulated an interest in research into the working conditions in the sector [3] and has led to more monitoring and increased pressure on MT organizations. The evidence accumulated in recent decades on the links between working conditions and the health and well-being of workers, as well as the relationships between the well-being of workers, productivity and quality of service [4] have generated considerable interest in the study of occupational risks in the MT sector in recent years.

The most prevalent risks are from the ergonomic risk of physical overexertion [5, 6] and from psychosocial factors [3, 7–11]. For both types of risk, a higher incidence of exposure among MT workers compared to other groups of the active population has been observed [3, 11]. With regard to working conditions, work content and how such work is organized, research to date has focused particularly on mental health problems arising from exposure to high stress situations and the emotional demands of MT [3, 11–13]. The sole focus of most of these studies is on detecting the risk factors for workers’ health, while few have analyzed the preventive role that might be played by variables that protect health or promote well-being.

Current research on the links between health and psychosocial risks at work in the MT sector provides little data on any differences in exposure to such risks there may be for people working in EMT and non-EMT settings. Neither is there any information in relation to other assignments in MT organizations, involving coordination or management activities. In research on working conditions and health, it is important to differentiate between sections of an organization – specifically, in this case, between the EMT and non-EMT services – in order to provide sufficient empirical evidence to allow informed decisions to be made within such organizations regarding the appropriateness of segmenting the allocation of workers to one service or another. At present, the functions of non-EMT professionals vary according to the MT model predominant in each country or even between regions or states of the same country [14, 15]. In some places, specialist patient retrieval services with dedicated mobile ICUs (Intensive Care Unit) are available alongside standard ambulance services [1, 16], with differently trained workers allocated to one service or the other, while elsewhere there is no specialisation at all and the same professionals perform both services [17]. There are also cases where personnel are on shifts rotating from one type of service to another [11]. Segmented data would also be very useful in designing the initial and life-long training of professionals in the sector regarding the prevention of occupational hazards.

Another problem with the research into the psychosocial risks faced by MT professionals and the effects on their health is the limited attention given to differences between genders – although some exceptions exist which appear to show more unfavourable results for women [18]. Taking into account the increased incorporation of women into the sector in recent years, both as drivers and as assistants [19], more studies are needed to provide evidence of whether the psychosocial risks and the health protective factors are experienced by men and women in different ways.

In view of the above, the main objective of this research is to analyze the differential exposure to various psychosocial risk and protective factors among MT workers according to their work setting (EMT, non-EMT and other jobs) and gender (men vs. women).

### Exposure to psychosocial risks to health and well-being among medical transport workers

Most studies on exposure to the psychosocial risks in MT and their influence on workers’ health and well-being focus mainly on stress [3, 7], posttraumatic stress [20, 21] and occupational burnout syndrome [3, 7, 8, 22]; other aspects investigated, to a lesser extent, include working atmosphere, work content, role clarity and conflict [7, 11] or shift work [23, 24]. According to the scientific literature reviewed, exposure to stressful working conditions or work setting does have an influence on the development of burnout [25–30]; for this reason, we have considered a combination of both working conditions and work setting in this study.

Occupational burnout is identified as a an altered emotional state which, according to Maslach and Jackson [31] can be evaluated via three ‘dimensions’: (i) *emotional exhaustion*, (EE) when the worker feels emotionally empty, exhausted by having to deal with demands that are overwhelming; (ii) *depersonalization* (DP) when the worker becomes insensitive to users of the service or the work being carried out, by treating them or addressing them in a cynical and dehumanized way and (iii) *reduced personal accomplishment* (PA), when the person experiences strong feelings of incompetence and failure. Although burnout studies generally focus on occupational health, this syndrome can also affect the well-being of people beyond the work environment [32]. The available data on MT workers is contradictory. While some studies point out that the probability of suffering from burnout is slightly higher for workers in MT than in other sectors of the active population [22], other research indicates that the probability is slightly lower [33].

With regard to working conditions, the data indicates that the severity of time pressure is a stress factor that can predict emotional exhaustion [34], together with the lack of control over the working environment [35], working in physically threatening environments [36] or the number of tasks per service [24]. With regard to the working environment, the lack of support among co-workers and/or superiors, the lack of communication and the intense pressure on personal relationships generated by the demands of the service, also had negative impacts on health [7, 37]. Similarly, shift work, which requires being awake when biologically one should be asleep, or role conflict, which involves contradictory demands or conflicts between one’s values ​​and the demands of work [7], are further examples of working conditions that have negative impacts on the health of MT professionals.

### Protective factors on health and well-being among medical transport personnel

Although most of the research to date on working conditions in MT has prioritized the analysis of psychosocial risk factors, some studies have emphasized the role of other variables that can have positive effects and act as protectors of health, such as the sense of coherence (SOC) [12, 38] and subjective well-being (SWB) [39].

A sense of coherence, or SOC, according to Antonovsky [40], is a global dispositional orientation ‘that expresses the extent to which one has a pervasive, enduring though dynamic feeling of confidence’ which is considered key to meeting the demands of the working environment and maintaining one’s health and subjective well-being. Antonovsky proposed that SOC can be evaluated through the components of comprehensibility, manageability and meaningfulness. Comprehensibility refers to a perception of a stressful stimulus – from one’s internal and external environments – in a structured, predictable and explicable way. Manageability implies that the resources required to meet the demands posed by these stimuli are available for the person to use. Meaningfulness refers to one’s perception of the stimulus. People with high SOC appear to cope better with stressful situations, are more flexible in selecting coping strategies, prioritize strategies that focus on the problem, and recover more quickly from stressful situations [41]. Studies in the context of MT have indicated that the SOC can act as a predictor of emotional exhaustion, depersonalization [42] and vulnerability to psychosocial risks [12]. Since MT personnel are highly exposed to critical events and their work is, emotionally, highly demanding, a high SOC may act as a protective factor against the effects of work stress and could act as a strategy to reduce the severity of symptoms resulting from psychosocial risks [38].

Subjective well-being, or SWB, refers to how people evaluate their lives, both at a general level and in specific areas of their lives (family, friends, free time, etc.). According to Cummins and Cahill [43], it is generally agreed that SWB is composed of three elements comprising a combination of one cognitive process (satisfaction /dissatisfaction judgments) and two affective processes (positive and negative affect). In the context of MT, although the literature includes studies showing low levels of SWB among MT personnel [7], there are also studies that indicate they have higher levels when compared to other professions such as fire fighters or police officers [44]. In the MT sector, SWB has been linked to other work factors: fatigue as a result of shift work was found to be a significant risk for SWB [45] while training to improve resilience at work acts positively on SWB [39].

With regard to working conditions, a positive work climate – which includes good communication, trust, social support and a feeling of belonging to the group – can act as a protective factor for health and well-being, helping to reduce the probability of burnout [46].

Although some of the studies reviewed above have investigated the impact of various psychosocial factors on the health and well-being of MT workers [7, 12], none of them examine whether the type of work being done and the gender of the individuals (and the interaction between them) influences MT workers’ exposure to the various indicators of risk/protection relating to psychosocial health. Information on this issue can facilitate decision-making in the organization and management of the staff in the companies carrying out these services, as well as the transfer of good practices from one service to another.

In light of the above, we set ourselves the following objectives:

1) To identify the exposure to various psychosocial indicators of risk and protection for health and well-being in a sample of medical transport professionals: working conditions, satisfaction with work shifts, emotional exhaustion, depersonalization, personal accomplishment, sense of coherence and subjective well-being.

2) To analyze the effect of work setting and gender on the various health-related risk/protection indicators.

3) To analyze the effect of the *interaction* between work setting and gender on the various health-related risk/protection indicators.

## Methods

The study population comprised the entire workforce of a company carrying out both EMT and non-EMT services in the province of Girona, Catalonia (Spain), at the time of the study. This workforce consisted of 438 medical transport professionals, of which 80.45% are men; 25.57% work in a non-EMT setting, 65.52% in an EMT setting (both basic life support and advanced life support) while 8.9% carry out group coordination activities, manage the coordinating centre or are in positions of responsibility for certain departments or areas of ​​the infrastructure. There is only one level of training for all medical transport professionals in Spain, the *ciclo formativo de grado medio* [47] (equivalent to a Vocational Education and Training certificate) for Emergency Health Personnel. This consists of approximately 2000 h of training, based on a programme developed during 2007, and is a required qualification for anyone, regardless of gender, seeking to work in either EMT or non-EMT services.

Participation in this study was voluntary and 217 (49.32%) questionnaires were returned, of which 16 were excluded due to lack of information on age and gender leaving a final sample of 201 (45.68%) professionals. The percentage of responses obtained was typical of these kinds of studies and was, in fact, higher than previous data-gathering efforts carried out in this same organisation. The final sample comprised 76.60% men, 21.80% non-EMT workers, 73.60% EMT workers and 4.60% other workers, thus reflecting approximately the same distribution of the workforce, in terms of gender and job position. The average age of respondents was 37.57 ± 7.15 (range 23 − 59) and the average length of service 8.75 ± 3.69 years (range: 1-27).

Working conditions were assessed using the *Escala de Clima Psicosocial en el Trabajo* [*Work Psychosocial Scale* (WPS-16)] developed by Tous, Bonasa and Mayor (2011) [48]. This scale provides a quick diagnosis on those working conditions that staff perceive as positive or negative in their organization. It is divided into three subscales: *personal relations* (PR), which involve self-esteem, social support and perceived integration into the social group; *work content* (WC), which is part of the set of stress factors relating to the individual’s work and *role definition* (RD) which is related to the perception of good management, supervision and job recognition [48]. The scale consists of 16 items with answers ranging from 1 (completely disagree) to 7 (completely agree). The Cronbach’s alpha for the present sample is 0.87 for the whole scale, and 0.74 in the validation sample.

Because there is a large disparity in the distribution of working days for the different jobs among the staff in our study, we included a question on their satisfaction with their schedule based on a scale of 1 (completely unsatisfied) to 10 (completely satisfied).

Burnout was explored using the Spanish version of the Maslach Burnout Inventory, MBI, [49] which consists of 22 items, each with a response scale of 0 (never) to 6 (daily). According to some of the studies consulted [50, 51], one or more exacerbated dimensions in the MBI is considered an indication of the presence of professional burnout. The internal consistency of the present sample was measured using Cronbach’s alpha, producing values ​​of 0.84 for emotional exhaustion, 0.50 for depersonalization and 0.82 for personal accomplishment, with the values ​​for the Spanish version being 0.90, 0.79 and 0.71, respectively.

The SOC among the respondents was explored using the Spanish version of the abbreviated Antonovsky’s Life Orientation Questionnaire [52] which consists of 13 items with a response scale of 1 (rarely or never) and 7 (very often), classified according to the three dimensions of SOC: comprehensibility, manageability and meaningfulness. Items with a negative direction were reversed before carrying out statistical analysis. Cronbach’s alpha for the present sample is 0.80 for the total scale, matching the Spanish version which was also 0.80.

Subjective well-being was explored using the Personal Welbeing Index, PWI, [53] as adapted by Casas et al. [54], which consists of 7 items, with a response scale of 0 (completely unsatisfied) to 10 (completely satisfied). The items refer to satisfaction with health, standard of living, life achievements, sense of security, the groups of people of which they form part, future security and relationships with other people. The analysis of internal consistency of the PWI for the present sample indicates a Cronbach’s alpha of 0.87; other studies have obtained values ranging between 0.70 and 0.85.

All the scales used have been validated for Spanish and have been employed previously in other studies which showed them to have satisfactory psychometric properties.

The questionnaire was handed out in 2014, in face-to-face meetings which included an explanation of the objectives of the study and the procedure to be followed. Each questionnaire was handed out with an envelope that could be sealed in order to guarantee the anonymity of the participants. With the same purpose in mind, it was explained that the questionnaires were to be handed in to area or department chiefs, who would then pass on the envelopes unopened to the person in charge of the study. During the period established for handing out the questionnaires, various reminders were issued with the aim of maximising participation.

Statistical analysis was carried out using the SPSS v.23 statistical software package. Descriptive statistics were used to analyze exposure to the various risk and protection factors considered (Objective 1). In order to analyze the main effects of gender (man vs. woman) and work setting (EMT, non-EMT and others) (Objective 2), and also to analyze the interaction between work setting and gender (Objective 3) on these workers’ exposure to the various risk and protection indicators (burnout, SOC, PWI, WPS-16 and satisfaction with schedule), we carried out a MANOVA test and applied Tukey post-hoc contrast to analyze the differences between groups. Since heterogeneity existed in the variances and covariances in the explored variables, according to the Levene test and Box test respectively, a more restrictive criterion was chosen following the recommendations by Olson [55], using the Pillai-Bartlett trace with a level of significance of less than 0.01 [56, 57].

## Results

The first objective was to identify the exposure of workers to the various risk factors and protection indicators regarding their health and well-being. Table 1 shows the results of the descriptive analysis performed for the different variables of each scale. The total mean score on the SOC scale is 70.07 with a standard deviation of 9.79. With regard to subjective well-being, as measured by the PWI, the item with the highest score for satisfaction is “life achievements” while the most negative was “future security”.

**Table 1:** Descriptive analysis of the following scales: WPS-16 satisfaction with schedule (R [1–10]), MBI, SOC and PWI (R[0-10])

Scale	Variable	Mean	Typical deviation	CI 95%
WPS-16	Work content	20.47	4.81	19.78-21.15
	Personal relations	34.95	4.54	34.31-35.60
	Role definition	33.75	6.18	32.86-34.65
Satisfaction	Schedule	7.38	2.78	6.99-7.77
MBI	Emotional exhaustion	10.51	9.015	9.20-11.81
	Depersonalization	4.24	4.27	3.64-4.85
	Personal achievement	41.02	7.55	39.91-42.12
SOC	Manageability	20.79	3.64	20.28-21.30
	Comprehensibility	25.88	4.84	25.20-26.56
	Meaningfulness	23.39	3.27	22.94-23.85
PWI	Health	8.05	2.10	7.76-8.34
	Standard of living	7.92	1.91	7.66-8.19
	Life achievements	8.28	1.59	8.06-8.50
	How safe you feel	8.24	1.76	7.99-8.49
	Groups of people you are part of	8.11	1.81	7.86-8.36
	Future security	6.41	2.47	6.07-6.76
	Relationships with other people	8.26	1.68	8.03-8.49
	Total	79.02	14.46	76.99-81.04

Note*. CI* Confidence interval, *WPS* Work Psychosocial Scale, *MBI* Maslach Burnout Inventory, *SOC* Sense of Coherence, *PWI* Personal Well-Being Index

In order to assess the risk of burnout, we calculated the response percentages for each of the three burnout categories (low, medium and high) corresponding to the EE, DP and PA scales, in accordance with the cut-off points proposed in the Spanish MBI [49]. For emotional exhaustion, the responses obtained were 74.2%, 17.2% and 8.6% for the low, medium and high categories respectively; for depersonalization, 55.9%, 32.3% and 11.8%; and for reduced personal accomplishment, 11.5%, 19.1% and 69.4%. It should be noted that in 67.6% of the cases (CI 95% of 42.3-86.9) there are one or more exacerbated dimensions of the MBI, with medium or high levels suggesting the presence of professional burnout.

With regard to the second objective, statistically significant differences in some of the risk/protection indicators were observed between the two work settings but not between genders (Tables 2). Those people working in non-EMT services have more unfavourable results regarding work content and satisfaction with schedule. They also exhibit more emotional exhaustion, less manageability and are less satisfied with their life achievements, with how safe they feel and with their future security than people working in EMT services.

The third objective was to analyze the interaction effect of work setting and gender on the dependent variables under consideration (i.e., the three WPS-16 dimensions, satisfaction with schedule, the three MBI dimensions, the three SOC dimensions and the seven items in the PWI). No significant interaction between the work setting and gender was found. On the other hand, work setting was able to explain the overall variability of the psychosocial factors considered (Tables 3 and 4).

**Table 2:** Analysis of variance in WPS-16, satisfaction with schedule, MBI, SOC and PWI for work setting and gender and the interaction between work setting and gender

Source	Scale	Dependent variable	Sum of squares	F	DF	Sig.
Work setting	WPS-16	Work content	533.09	12.87	2	0.00
		Personal relations	81.69	40.84	2	0.12
		Role definition	184.21	2.63	2	0.07
	Satisfaction	Schedule	327.44	12.87	2	0.00
	MBI	Emotional exhaustion	707.27	4.73	2	0.01
		Depersonalization	23.28	0.76	2	0.46
		Personal achievement	20.92	0.81	2	0.81
	SOC	Manageability	139.41	5.48	2	0.00
		Comprehensibility	90.64	1.82	2	0.16
		Meaningfulness	54.21	2.29	2	0.10
	PWI	Health	15.82	1.67	2	0.19
		Standard of living	8.33	1.03	2	0.35
		Life achievements	28.61	5.65	2	0.00
		How safe you feel	27.72	4.56	2	0.01
		Groups of people you are part of	4.41	0.65	2	0.52
		Future security	38.79	3.50	2	0.03
		Relationships with other people	9.71	1.62	2	0.20
Gender	WPS-16	Work content	21.77	1.05	1	0.30
		Personal relations	7.19	0.37	1	0.53
		Role definition	31.27	0.89	1	0.34
	Satisfaction	Schedule	1.53	0.35	1	0.55
	MBI	Emotional exhaustion	37.35	0.50	1	0.48
		Depersonalization	40.97	2.69	1	0.10
		Personal achievement	0.32	0.00	1	0.93
	SOC	Manageability	3.94	0.31	1	0.57
		Comprehensibility	10.94	0.44	1	0.50
		Meaningfulness	0.20	0.02	1	0.88
	PWI	Health	3.07	0.65	1	0.42
		Standard of living	0.03	0.00	1	0.92
		Life achievements	1.00	0.39	1	0.52
		How safe you feel	0.09	0.03	1	0.86
		Groups of people you are part of	0.95	0.28	1	0.59
		Future security	0.187	0.03	1	0.85
		Relationships with other people	1.88	0.62	1	0.42
Work setting*gender	WPS-16	Work content	36.34	0.87	2	0.41
		Personal relations	5.79	0.15	2	0.85
		Role definition	8.18	0.04	2	0.95
	Satisfaction	Schedule	3.30	0.38	2	0.68
	MBI	Emotional exhaustion	52.48	0.35	2	0.70
		Depersonalization	5.32	0.17	2	0.84
		Personal achievement	94.91	0.90	2	0.40
	SOC	Manageability	33.75	1.32	2	0.26
		Comprehensibility	5.62	0.11	2	0.89
		Meaningfulness	9.29	0.47	2	0.62
	PWI	Health	3.51	0.37	2	0.69
		Standard of living	0.91	0.088	2	0.91
		Life achievements	2.23	0.44	2	0.64
		How safe you feel	5.09	0.83	2	0.43
		Groups of people you are part of	7.2	1.06	2	0.34
		Future security	5.87	0.53	2	0.59
		Relationships with other people	1.85	0.31	2	0.73

Note. *WPS* Work Psychosocial Scale, *MBI* Maslach Burnout Inventory, *SOC* Sense of Coherence, *PWI* Personal Well-Being Index

*p value < .05

**Table 3:** Multivariate analysis of variance in WPS-16, satisfaction with schedule, MBI, SOC and PWI for work setting and gender

Effect	Value	F	DF	Error df	Sig.
Work setting	0.64	3.48	34.00	250.00	0.00
Gender	0.06	0.51	17.00	124.00	0.94
Work setting*Gender	0.15	0.61	34.00	250.00	0.95

Note*. WPS* Work Psychosocial Scale, *MBI* Maslach Burnout Inventory, *SOC* Sense of Coherence, *PWI* Personal Well-Being Index

* *p* value < .05

**Table 4:** Multivariate Analysis according to work setting

Dependent variable (statistically significant)	Work setting		Value	Std. Error	Sig.	CI 95%	
Work content	EMT	non-EMT	4,86*	0,91	0,00	2,69	7,04
		Others	-1,21	1,77	0,77	-5,42	2,99
	non-EMT	EMT	−4.86*	0.91	0.00	−7.04	−2.69
		Others	−6.08*	1.89	0.00	−10.57	−1.58
	Others	EMT	1.21	1.77	0.77	−2.99	5.42
		non-EMT	6.08*	1.89	0.00	1.58	10.57
Role definition	EMT	non-EMT	3.24*	1.19	0.02	0.42	6.07
		Others	1.53	2.30	0.78	−3.93	7.00
	non-EMT	EMT	−3.24*	1.19	0.02	−6.07	−0.42
		Others	−1.71	2.46	0.76	−7.56	4.14
	Others	EMT	−1.53	2.30	0.78	−7.00	3.93
		non-EMT	1.71	2.46	0.76	−4.14	7.56
Satisfaction with Schedule	EMT	non-EMT	4.12*	0.41	0.00	3.13	5.11
		Others	2.07*	0.80	0.03	0.16	3.98
	non-EMT	EMT	−4.12*	0.41	0.00	−5.11	−3.13
		Others	−2.05*	0.86	0.04	−4.10	−0.01
	Others	EMT	−2.07*	0.80	0.03	−3.98	−0.16
		non-EMT	2.05*	0.86	0.00	0.01	4.10
Emotional exhaustion	EMT	non-EMT	−5.77*	1.74	0.00	−9.89	−1.64
		Others	−6.87	3.37	0.10	−14.85	1.11
	non-EMT	EMT	5.77*	1.74	0.00	1.64	9.89
		Others	−1.10	3.60	0.95	−9.64	7.44
	Others	EMT	6.87	3.37	0.10	−1.11	14.85
		non-EMT	1.10	3.60	0.95	−7.44	9.64
Manageability	EMT	non-EMT	2.16*	0.71	0.00	.46	3.86
		Others	1.37	1.39	0.58	−1.93	4.66
	non-EMT	EMT	−2.16*	0.71	0.00	−3.86	−.46
		Others	−0.79	1.48	0.85	−4.32	2.73
	Others	EMT	−1.37	1.391	0.58	−4.66	1.93
		non-EMT	0.79	1.48	0.85	−2.73	4.32
Life achievements	EMT	non-EMT	0.75*	0.32	0.20	0.12	1.39
		Others	1.39*	0.62	0.27	0.16	2.62
	non-EMT	EMT	−0.75*	0.32	0.02	−1.39	−0.12
		Others	0.64	0.66	0.33	−0.67	1.95
	Others	EMT	−1.39*	0.62	0.02	−2.62	−0.16
		non-EMT	−0.64	0.66	0.33	−1.95	0.67
How safe you feel	EMT	non-EMT	.61	0.35	0.19	−0.22	1.44
		Others	1.80*	0.68	0.02	0.19	3.41
	non-EMT	EMT	−0.61	0.35	0.19	−1.44	0.22
		Others	1.19	0.72	0.23	−0.53	2.91
	Others	EMT	−1.80*	0.68	0.02	−3.41	−0.19
		non-EMT	−1.19	0.72	0.23	−2.91	0.53
Future security	EMT	non-EMT	0.42	0.47	0.64	−0.70	1.55
		Others	2.19*	0.91	0.04	0.02	4.37
	non-EMT	EMT	−0.42	0.47	0.64	−1.55	0.70
		Others	1.77	0.98	0.17	−0.55	4.10

Note. *CI* Confidence interval, *EMT* Emergency Medical Transport, *Non-EMT* Non-Emergency Medical Transport

* p value < .05

## Discussion

The results obtained in relation to exposure to various factors of risk and protection for health and well-being (i.e., the first objective), show that work content, personal relations and role definition are more favourable for MT workers than the reference values ​​in the instrument validation study [48]. With regard to burnout, EE, DP and PA, these are more favourable among the MT workers in our study than the mean values of the Spanish reference sample [49] as well as those of other previous studies in the MT sector [50]. However, 67.66% of workers in our study have one or more exacerbated dimensions, which is indicative of burnout. Although this figure is lower than the 88.8% obtained in [50], it remains important enough to require action aimed at reducing it. With regard to the SOC results, the mean score obtained by the participants in our study is slightly higher than the mean score obtained for a sample of nursing professionals working in the same Girona district [58] and is very similar to the mean score for a sample of MT professionals in Sweden [12]. No other studies on SOC in the Spanish MT sector have been found. The scores for personal well-being are within the normative values ​​defined for Western countries. What is noteworthy is the low score for the respondents’ satisfaction with their future security, 6.42 ± 2.48 (95% CI 6.07-6.76). This may be due to the fact that MT management contracts are awarded through public tender which entails the possibility of companies changing from time to time, leading to some uncertainty as far as job security is concerned. Other studies also indicate that scores for future security tend to be lower, compared to other fields, among professionals aged between 36 and 45 [59].

Regarding the analysis of the different work settings (Objective 2), it was observed that, in all cases, the results are more unfavourable for workers in non-EMT services than for those in EMT services. Furthermore, the mean scores for professionals performing other jobs in the MT sector were sometimes even more unfavourable than those of the non-EMT workers. We will now give some possible explanations for these results. Different levels of satisfaction with work content, for example, appear to be the result of the tasks required by each type of service. Non-EMT tasks are more repetitive and less diverse than EMT tasks and, in addition, may sometimes be carried out repeatedly along the same routes over the course of many days. For EMT workers, however, each service is different and occurs in a different place each time. Furthermore, the work in the each setting is organized in different ways. Non-EMT tasks are organized by a coordination centre to take place throughout the day, one after the other without interruption. The situation is markedly different for EMT workers who spend their working day at the base ready to respond to requests for emergency services. The difference in satisfaction levels with regard to schedule may be due to the system of shifts established by the management of the service: EMT shifts are typically 24-h days every 3 or 4 days, while non-EMT working hours involve different 8 or 10-h shifts for 4 or 5 days a week. The different working hours are more favourable to EMT workers, providing them with more quality time with their families, more free time and more time for training or educational opportunities, among others. As for emotional exhaustion, the differences observed between EMT and the non-EMT workers could be due to a greater sense of worth they feel in their work among the EMT workers which may act as a protective factor [11]; similarly, the sense of worth at work may also explain the different scores observed in sense of coherence or subjective well-being [38]. The different scores for future security could be explained either by work schedule or reduced chances of promotion. Firstly, in the case of the work schedules among this study group, EMT schedules commonly remain stable from one year to the next, while non-EMT schedules are subject to frequent modifications, which may have a negative impact on the sense of future security for non-EMT workers. Secondly, internal promotion: all MT professionals in Spain, regardless of whether they perform EMT or non-EMT tasks [60], must be in possession of the mid-level training certificate for Emergency Health Personnel [47]. This means that there are a large number of non-EMT professionals who aspire, eventually, to be able to work with the EMT service; thus there is considerable competition for places which has a negative impact on their chances of promotion. Since the training is the same for both EMT and non-EMT staff, this should not, a priori, have any influence on the results. However, the fact that only one type of training exists among all MT personnel may generate expectations of non-EMT staff taking on EMT tasks and vice versa.

As for the third objective, the results show that there is no significant interaction between the work setting and gender. This result seems to indicate that the health-related risk/protection factors affect men and women in similar ways in both work settings, EMT and non-EMT. This is in contrast with previous studies which have indicated a greater impact of the psychosocial risk on the health of women [18].

The relationships observed between the risk and protective factors explored, suggest action should be taken to improve working conditions and to strengthen the sense of coherence and well-being among medical transport professionals as factors that protect against occupational burnout syndrome. Efforts to enhance working conditions should concentrate on the workers’ perceptions in pursuit of improvement measures that promote a climate conducive to health and well-being. In order to strengthen the SOC, ongoing training programmes could be implemented annually in the organization’s training plans and mutual help groups can be set up to share professional and/or personal experiences. Likewise, subjective well-being would benefit from plans to promote healthy habits and lifestyles.

This kind of study has certain limitations which need to borne in mind. Firstly, the results are obtained by self-report and depend, therefore, on the degree of sincerity among the participants who work for the company in which the study was carried out. It was for this reason that we decided not to compromise the anonymity of the participants by including any further segmentation of the demographic variables (e.g., work zone, basic and advanced life support, etc.). Secondly, it is not known whether those workers who declined to participate would have obtained similar scores in the questionnaires to those who did participate. Finally, the low reliability of the *depersonalization* dimension in the MBI scale needs to be taken into account and caution is advised in the interpretation of the results, although, in fact, no significant results for depersonalization were observed in this study.

## Conclusions

To the best of our knowledge, the present study is the first to link working conditions with the constructs of sense of coherence, personal well-being and occupational burnout syndrome in the medical transport (MT) sector while also identifying the differential effects of these factors on workers according to work setting (EMT vs. non-EMT) and gender. The fact that we obtained statistically significant differences between EMT and non-EMT personnel in some of the variables analyzed raises important questions for all those organizations that have been awarded the public tender of both services, and have chosen to separate the two services, assigning their workers to one service or the other, without transfer options between the two. The question arises, therefore, as to whether it is preferable to keep each service separate or whether the workers should be involved in both types of service in order to prevent the symptoms of burnout found among the non-EMT staff. For example, as a preventative measure, Jonsson [12] suggested that in order to reduce the effects of posttraumatic stress symptoms among EMT personnel, these workers could perform tasks in the non-EMT service for a certain period. Both EMT and non-EMT personnel perform important work in the public health chain and it is therefore equally important to take care of them. Hence the need for detailed information on the current situation in terms of their exposure to risks derived from work, as well as those factors protective of their health and well-being. Further investigation is needed into the working conditions in the EMT and non-EMT services to find the divergent aspects that could explain the results found in our study. In this sense, we consider that the data obtained constitutes an important and useful knowledge base for future lines of research or for implementing and promoting good practice in prevention and training in the sector.

Among the future lines of research to be considered, we suggest: 1) further analysis of risk-protective factors for health and well-being from the perspective of the professionals in the sector, paying special attention to their own explanations of the differences between working conditions in the EMT and the non-EMT services; 2) a comparative analysis of the various professional MT training programmes at international level in order to assess the content provided by formal training and what could be usefully added to this training, both in terms of reducing risk factors and promoting protective factors for health and well-being; and 3) an investigation into whether the training qualifications for each service (EMT and non-EMT) should be the same, as is the case in some countries, or different, as is the case elsewhere.

## Abbreviations

CI: Confidence Interval
DP: Depersonalization
EE: Emotional Exhaustion
EMT: Emergency Medical Transport
ICU: Intensive Care Unit
MBI: Maslach Burnout Inventory
MT: Medical Transport
Non-EMT: Non-Emergency Medical Transport
PA: Personal Accomplishment
PR: Personal Relations
PWI: Personal Well-Being Index
RD: Role Definition
SOC: Sense of Coherence
SPSS: Statistical Package for the Social Science
SWB: Subjective Well-Being
WC: Work Content
WPS: Work Psychosocial Scale
